# Primary Care Practitioners’ Barriers to and Experience of COVID-19 Epidemic Control in China: a Qualitative Study

**DOI:** 10.1007/s11606-020-06107-3

**Published:** 2020-08-31

**Authors:** Zhijie Xu, Yuanqu Ye, Yang Wang, Yi Qian, Jianjiang Pan, Yiting Lu, Lizheng Fang

**Affiliations:** 1 Department of General Practice, Sir Run Run Shaw Hospital, Zhejiang University School of Medicine, Hangzhou, 310016 Zhejiang China; 2 Baili Community Healthcare Center, The People’s Hospital of Longhua, Shenzhen, China; 3 Chinese General Practice Press, Beijing, China

**Keywords:** COVID-19 epidemic, primary care practitioners, qualitative study, China

## Abstract

**Background:**

The coronavirus disease 2019 (COVID-19) emerged in December 2019 and posed numerous challenges to China’s health system. Almost 4 million primary care practitioners (PCPs) participated in controlling the outbreak. However, PCPs’ barriers to and experience of the epidemic control remain unknown and are essential for improving countermeasures.

**Objective:**

To better understand the barriers PCPs faced in COVID-19 epidemic control and their psychological and occupational impacts, and explore potential solutions.

**Design:**

This qualitative study was conducted through semi-structured, in-depth interviews from February 12, to March 10, 2020.

**Participants:**

A purposive sample of frontline PCPs affiliated with either community health centers or township health centers in four provinces of China were recruited.

**Approach:**

Interviews were conducted by telephone, and then recorded, transcribed, and content analyzed. Themes surrounding PCPs’ barriers to COVID-19 epidemic control, their experience, and potential solutions were iteratively identified using the constant comparative method.

**Key Results:**

Of the 21 PCPs interviewed, 10 (48%) were women and 5 (24%) worked in rural areas. Barriers to epidemic control in primary care included inappropriate PCP scheduling and role ambiguity, difficult tasks and inadequate capacities, and inexperienced community workers and insufficient cooperation. Some PCPs perceived respect and a sense of accomplishment and were preoccupied with the outbreak, while others were frustrated by fatigue and psychological distress. PCPs reported potential solutions for improving countermeasures, such as improving management, optimizing workflows, providing additional support, facilitating cooperation, and strengthening the primary care system.

**Conclusions:**

Due to their roles in controlling the COVID-19 epidemic, PCPs in China faced a series of barriers that affected them physically and mentally. Support for PCPs should help them to overcome these barriers and work efficiently. The current findings provide insight into the challenges and potential solutions for strengthening the preparedness and response of China’s primary care system in future disease outbreaks.

**Electronic supplementary material:**

The online version of this article (10.1007/s11606-020-06107-3) contains supplementary material, which is available to authorized users.

In December 2019, a novel coronavirus was detected from a series cases of pneumonia of unknown cause in Wuhan, China, which was subsequently named the coronavirus disease (COVID-19) by the World Health Organization (WHO).^1–3^ As of June 13, 2020, 84,671 cases including 4645 deaths had been confirmed in China,^4^ and a global pandemic had emerged. In addition, a total of 215 countries had reported over 7,640,000 confirmed cases and 426,000 deaths, and the numbers continue to grow.^4–6^

Primary care practitioners (PCPs) are essential in confronting the pandemic. For example, almost 4 million PCPs in China participated in COVID-19 epidemic control.^7^ They worked in collaboration with the community workers and community police in a “Joint Defense Team” led by the neighborhood committee.^8^ PCPs were responsible for screening suspected cases, visiting residents in quarantine, contact tracing and monitoring, and surveillance at checkpoints, while community workers and community police provide non-medical support to residents in quarantine.^8^ PCPs regularly recorded work-related information on forms and uploaded the forms to the neighborhood committee and health administrative departments. Similar procedures were undertaken by PCPs in Singapore.^9^

Numerous studies have focused on barriers to epidemic control in primary care. A systematic review identified challenges faced by PCPs in different countries during previous pandemic response, such as a shortage of personal protective equipment (PPE), limitations of provided information, and insufficient training.^10^ A qualitative study showed that PCPs experienced difficulties in translating pandemic guidelines into practice.^11^ However, the barriers PCPs encountered in COVID-19 epidemic control and their solutions have not been explored.

Another research focus has been the impact of epidemic control on PCPs and their experiences. A recent survey showed that general practitioners (GPs) in Shanghai displayed psychological health problems of varying severity during epidemic control, and 88.4% of GPs felt stressed.^12^ Similarly, previous studies demonstrated that the pandemic outbreak changed PCPs’ work environment and lifestyle,^13^ and led to a series of negative emotions, such as depression, anxiety, and fear.^14^ They could also experience symptoms of acute stress disorders or post-traumatic stress disorder (e.g., intrusion, avoidance, and hyperarousal) after the outbreak.^15^ Therefore, PCPs’ experience in COVID-19 epidemic control deserves closer attention to inform improvement efforts.

To understand PCPs’ perceived barriers to and experience of performing their tasks in epidemic control, we recruited frontline PCPs in China and conducted in-depth interviews using a qualitative design. We aim to understand PCPs’ perspectives on their work and explore the strategies for improving countermeasures in primary care.

## METHODS

### Design and Study Setting

From February 12 to March 10, 2020, we conducted a descriptive qualitative study involving semi-structured, in-depth interviews with purposive samples of PCPs.^16^ Interviews were conducted by telephone because of the nationwide traffic restriction, and they lasted a mean of 34 minutes (range: 30–45 minutes). All participants provided verbal informed consent before the interviews began and were not compensated for their participation. The study was approved by the Sir Run Run Shaw Hospital Ethics Committee and adhered to the Declaration of Helsinki.^17^

### Participants

We used WeChat, an instant messaging app, to invite PCPs to participate in the interviews, using the principle of maximum variation.^18^ Three family physicians refused to participate because they were not responsible for tasks in epidemic control. Participants were affiliated with local government–owned community health centers in urban areas (Zhejiang and Guangdong province) or township health centers in rural areas (Shaanxi and Hunan province). Participants knew the investigators prior to the interview, but none had worked with the investigators. The sample size was determined using thematic saturation: two investigators (Z.X. and Y.Y.) analyzed the transcripts and notes for newly emergent themes after the first 10 in-depth interviews, and after every 1 or 2 thereafter. We stopped scheduling interviews when additional interview data created little or no change to the codebook and no new patterns or themes emerged.^19, 20^ Repeat interviews were not carried out.

### Interview Guide

The interview guide was adapted from relevant qualitative studies involving healthcare workers in infectious disease outbreak,^21, 22^ and was refined through pilot interviews with three PCPs to improve appropriateness and clarity (eAppendix 1). Each interview began with a question about the types of tasks participants had performed in epidemic control. Probing questions were then used to encourage participants to describe tasks in which they felt their performance was deficient and whether they encountered any barriers to task performance (e.g., how did the barriers or difficulties affect your work?). Probing questions also elicited details of PCPs’ experiences and the occupational and psychological effects of epidemic control (e.g., have you experienced any positive or negative emotion?). At the end of the interviews, investigators encouraged PCPs to talk freely about their perspectives regarding strategies that could contribute to improved control measures in primary care.

### Data Collection and Analysis

Information regarding participants’ characteristics was collected before the interviews, which were independently audio recorded and transcribed verbatim by two male general practitioners as interviewers (Z.X. and Y.Y.) who had received training on qualitative interviewing. The interviewers made field notes during the interview when necessary. They independently identified major themes and subthemes via thematic content analysis and developed a preliminary codebook for data analysis based on the first three transcripts.^23^ They reviewed transcripts continuously using the constant comparative method to expand existing themes and identify new ideas or themes.^24^ The codebook was iteratively refined and finalized via internal consensus until 100% agreement was reached. MAXQDA (version 2018.1.1) was used in the data analysis and retrieval.

Transcripts were not returned to participants for comment or correction, but we randomly selected three participants and sent them our main findings via e-mail. They agreed with the themes without modification.

## RESULTS

We recruited 21 eligible PCPs (14 family practitioners, 4 internists, 2 surgeons, and 1 pediatrician) from 21 practices (16 community health centers and 5 township health centers). Of the 21 participants, 10 (48%) were women, and 5 (24%) participants undertook administrative tasks in their medical practice. The mean age of participants was 36 years (range: 29–46 years), and the mean duration of practice was 12 years (range: 3–25 years) (eAppendix 2).

### Barriers to Outbreak Control in Primary Care

#### Inappropriate PCP Scheduling and Role Ambiguity

Participants described numerous barriers to epidemic control (Table 1). Some felt overburdened and assigned to unsuitable positions. One family physician explained this feeling using a surprising example: “There were 270 residents [to be quarantined] that day, but the community health center only assigned 5 physicians [to visit them].” Others felt confused, as they were asked by the leaders of their community/township health centers to perform low-skilled work and believed they needed an additional supportive workforce. The confusion was described by an internist as follows: “When I came back [from the home visits], I was not free until I disinfected the ambulance. But why not employ a cleaner?”

**Table 1:** **Barriers of Epidemic Control in Primary Care**

Barriers	Quotation
Inappropriate PCP scheduling and role ambiguity	
Improper task allocation	“The male physicians were allocated to the checkpoint overnight. They are very tough.”
Inflexible policy	“In my community, residents born in Hubei, coming from or via Hubei were uniformly quarantined at home regardless of symptoms.”
Excessive inspection and meetings	“I attended the meetings almost every two days. The meetings usually last an hour or two, but I need to make preparations before the meetings or inspection.”
Ambiguous instructions	“…and the guidance [of epidemic control] lacks detail and fails to assign clear responsibilities to the specific group or person. Sometimes, even the supervisors could not give us a definite instruction.”
Difficult tasks and inadequate capacities	
Overwork	“I work for almost 12 hours a day and have no day off…if one case was confirmed, then his neighbors living in the whole building, maybe a thousand people, would be quarantined. It required all physicians [from our institution] to visit.”
Complex task	“One [quarantined] resident called me at 10:00 pm asking whether she had [had a] stroke. She was really worried and contacted me at any time.”
Deficiency of workforce	“[It took me] a lot of time to visit the quarantined residents and no one could help me to analyze the data. I hope the paperwork could be specially assigned to someone.”
Lack of support	“I walked to visit the [quarantined] residents only with a medical mask. No gowns or goggles.”
Inexperienced community workers and insufficient cooperation	
Incapacity of community workers	“Nominally, our work is led by the committee of community; however, it is we physicians that guide community workers to control the outbreak because they always turned to us for help if anything new emerged.”
Inactiveness of community workers	“When I warned [a community worker] that he was mistakenly measuring the body temperature, he still went his own way perfunctorily…they seemed careless to the work.”
Work gap	“The communication [with community workers] of work was not running smoothly at the start [of epidemic control]. They seldom informed us of their next step.”

Some policies were considered inflexible and PCPs felt they limited effective scheduling. For example, the quarantine duration was fixed for everyone leaving the epidemic area, regardless of whether self-quarantine had already been undertaken. One participant commented, “[There is] a one-size-fits-all approach that we need to follow, and it took much more time to address the consequences.” These inflexible policies not only increased the unnecessary workload but also may have reduced residents’ trust in epidemic control.

Another reported barrier was excessive inspection and meetings. The government officials and medical experts irregularly visited the community/township health centers and inspected PCPs’ daily practice of epidemic control, including the material preparation and arrangement, and held meetings to discuss the existing problems and potential solutions with PCPs. One participant stated, “It really troubled me that I had to accompany those supervisors, maybe 3 to 5 times a week, and show them what we had done with countless papers and forms and photos.” Some instructions distributed to PCPs by supervisors were perceived as “scratching the surface”. In addition, the frequent modification of guidance regarding epidemic control confused PCPs.

#### Difficult Tasks and Inadequate Capacities

Although routine care was largely canceled in many primary care practices, participants frequently noted the deficiencies of the workforce and that they worked for extended hours during epidemic control. PCPs were on call 24 hours per day to visit newly quarantined residents. Online consultation with residents increased workload during time off. One family physician stated, “I often kept an eye on my mobile phone because the residents often left a message of inquiry in the WeChat group waiting for my reply.”

All participants had limited experience in working during a pandemic and more than half perceived their professional training as inadequate and not tailored to their work of epidemic control in the community. In addition, most institutions lacked PPE (particularly masks and gowns) and PCPs generally compromised their safety by reusing PPE.

#### Inexperienced Community Workers and Insufficient Cooperation

PCPs in China performed home visits for quarantined residents in cooperation with community workers.^8^ A few participants complained that community workers were sometimes inactive in terms of participation in epidemic control: “The home visits should be implemented by a group of family physicians and community workers, but sometimes they arrived late.” Another barrier PCPs encounter was that community workers received inadequate training in epidemic control. As one internist commented, “The temporarily recruited [community workers] had no clinical background; they might fall in a rut and fail to deal with things case by case.”

Participants also described a lack of cooperation between PCPs and community workers. One family physician stated, “It might cause a delay if there was any error of communication. Sometimes the community workers isolated the resident 1 or 2 days before I received the notice. But [the resident] still needed to stay at home for 14 days.” A possible explanation for the miscommunication was that orders were released by different administrative departments with limited previous interaction or experience of cooperation.

### Impact of Epidemic Control on PCPs

#### Preoccupation

The heavy tasks and work stress involved in epidemic control resulted in PCPs devoting additional energy and effort to their daily practice. One source of PCPs’ preoccupation with epidemic control was the culture of commitment and sacrifice in the healthcare workforce. One participant expressed his pride in participating in epidemic control: “In my school days, I witnessed the outbreak of SARS in 2003 and was impressed by the sacrifice of angels in white…so I feel proud to have the opportunity to control the outbreak on the frontline.”

#### Sense of Respect and Accomplishment

All participants expressed a sense of respect for epidemic control, which enhanced their relationship with residents. PCPs received increased emotional support and appreciation, and their efforts were recognized by residents in the community. Participants also expressed satisfaction with the insurance and compensation provided by the government. Some PCPs felt a sense of accomplishment when the quarantine expired, and the residents they managed were not infected. Others were inspired to have greater solidarity with colleagues, described as follows: “It’s impressive to see my colleagues bearing the hardship…our cohesion is greater than before and makes it all worthwhile.”

#### Fatigue

More than half of the participants claimed they experienced fatigue as a result of participating in epidemic control. Some participants complained that the work content was beyond their capacity and the requirements were incongruous with their training. The intensive work and tough tasks were described as the main factors affecting fatigue. Insomnia was cited as another cause of fatigue. One participant reported that the overwhelming work stress deteriorated his sleep quality, which led to inadequate rest and intensified his experience of fatigue. Many other symptoms, such as “memory decline,” “weight loss,” and “inappetence,” were reported as common concomitant manifestations of fatigue.

#### Psychological Distress

Participating in epidemic control made PCPs a vulnerable group susceptible to psychological distress (Table 2). Some experienced fear of being infected, and this fear was intensified by the inadequate supply of PPE and prolonged frontline work. PCPs frequently experienced anxiety because they needed to adapt to a fast-paced, highly efficient working environment. Some participants felt anxious about errors of omission and residents’ complaints. Most PCPs experienced frustration with the paperwork required for reporting, which was deemed as time-consuming but scarcely conducive to practice. Another reason for their frustration was that their efforts and contributions were not always recognized by supervisors. PCPs would become angry when residents refused to comply with the quarantine and were offended by scurrilous remarks. However, all participants denied persistent or severe depressive symptoms (e.g., feeling hopeless or suicidal thoughts).

**Table 2:** **Impact of Outbreak Control on PCPs**

Impact	Quotation
Preoccupation	“I was very cautious in my outpatient office and spent more time on patients who came to me for consultation. I do not want any potential cases omitted [female, family physician in community health center].”
Sense of respect and accomplishment	“When they were released from the quarantine and said ‘thank you, doctor’, I felt their sincere respect and gratitude [female, family physician in community health center]”
Fatigue	“I devoted to working day and night without a full day off…I slept three hours last night, and continued to work until thirty minutes ago…I felt endless exhaustion.”
Psychological distress	
Fear	“I understand the risk of infection is controllable, but what if I passed the infection to my family?”
Anxiety	“I was constantly taking on new tasks and adapting to new requirement, dealing with things that might come up. I was very anxious at that time.”
Frustration	“The guidance was problematic at the early stage of epidemic control, but we had no voice to make a change…I felt helpless and powerless.”
Anger	“Some villagers were frightened of virus transmission through us physicians and hurled insults at me…I choke down their acrimony…it’s very annoying.”

Most participants found psychological support from their colleagues for their psychological distress, but all participants described a lack of external support, and the reasons were “no available professional psychological support,” “too busy to seek for help,” and “won’t help things at all.”

### Potential Solutions

#### Improving Management and Supervision

To solve certain problems, such as improper task allocation and inflexible policies, participants generally suggested that administrative departments should develop measures that were more person-centered and based on specific contexts. One participant stated, “I appreciate those officials who listened to our voices, sympathized with our dilemmas, and were capable of providing practical strategies.” Moreover, streamlining excessive inspection and meetings was suggested by some participants. One surgeon remarked, “…facing a succession of inspections…then I became unmoved. I didn’t care about what they asked anymore.”

#### Optimizing Workflow

Many participants expressed a wish to work efficiently. For example, paperwork and reporting were frequently mentioned as a barrier to epidemic control and occupied much of PCPs’ time. Strategies involved rational workforce arrangement and internal coordination. As one participant stated, “[The medical institution] could recruit full-time medical assistants to perform the low-skilled tasks, such as the daily statistical report.” In addition, participants proposed the option of streamlining the procedures for reporting using an intelligent approach. One physician admitted, “The task of surveillance [at the checkpoints] is getting easier because now we have an identity database to screen the contact history for the travelers.”

#### Providing Necessary Support

Most participants emphasized an imperative of increasing the supply of PPE to PCPs, although they all understood the shortage.^25^ One suggested strategy was to “use PPE in a planned way” to ensure the security of the frontline healthcare workforce. Other options included “collecting PPE from the public” and “centralized purchasing.” Some participants thought they lacked the experience of coping with major infectious diseases and needed more professional training. One participant commented, “The online education program helped me gain much knowledge of COVID-19, but we need lessons more tailored for primary care.”

#### Facilitating Cooperation

Participants described the need to reinforce cooperation with community workers. Participants noted that it was necessary to identify the division of responsibilities for both sides and strengthen the training and supervision of cooperation. An effective approach would be to establish a mechanism of interaction and communication. As one participant stated, “Tacit cooperation cannot be expected in one stroke…if the effect of communication was not significant, then we must try again.”

#### Strengthening the Primary Care System

Participants unanimously agreed that the COVID-19 outbreak was a challenge to the Chinese primary care system. To strengthen this system, participants’ recommendations ranged from “increasing investment in primary care institutions” and “developing information technology” to “improving the capacity of healthcare personnel.” Participants expected a system that was “more resilient”, “offered universal coverage down to the community level,” and “provided integrated care for residents.”

## DISCUSSION

Primary care is the first line of defense in controlling an epidemic at a community level, but the susceptibility of PCPs to tasks and the serious consequences were not fully recognized. To enhance understanding of the current status of COVID-19 epidemic control in primary care, we characterized PCPs’ perceived barriers and experience. We also examined PCPs’ perspectives on the solutions that could potentially benefit the primary care system in coping with major infectious diseases.

To our knowledge, this was the first qualitative study to explore PCPs’ work in major infectious disease control in China. The PCPs described a series of barriers to epidemic control. Aside from the extreme workload, rapidly evolving practice environment, PPE shortage, and inadequate training, which are consistent with international reporting,^26, 27^ participants emphasized specific concerns about inappropriate PCP scheduling and role ambiguity, which complicated their routine work, and insufficient cooperation with community workers, which reduced their work efficiency. These findings highlight new problems within and beyond the primary care system during emergency emergencies. Therefore, a feedback channel between PCPs and leaders should be established to detect problems in epidemic control.

Implementation of epidemic control had varied occupational and psychological effects on PCPs. Some PCPs responded to the epidemic proactively because of inner motivation or external pressure, whereas others felt fatigued and expressed psychological distress. Evidence suggests that frontline healthcare workers are generally vulnerable to the emotional impact of epidemics.^28–30^ In this study, we inductively identified 4 manifestations of psychological distress among participants—fear, anxiety, frustration, and anger (Table 2)—most of which were reported as mild in degree and short in duration, and seldomly the cause of absenteeism or disease. Our findings provide insights into the factors affecting emotions that primary care managers should acknowledge. For example, PCPs felt frustrated with the paperwork of reporting surveillance data not only because it was time-consuming or complex but also it was of little practical value. Remarkably, most participants found support from their colleagues, but none received external psychological support, suggesting potential gaps in mental health services for PCPs during emergencies.^31^ Although many studies have reported that positive professional relationships, including dialogue and emotional support, were an essential protective factor for preventing physician burnout,^32^ our findings support and expand on the existing knowledge regarding the essential role that the peer support plays in PCPs’ psychological support during a time of pandemic and workforce scarcity.

Strategies to help PCPs overcome challenges and prevent the primary care system from being overwhelmed are urgently needed. First, health authorities and institutional leaders were expected to provide specific support in terms of material, technology, and mental care to make PCPs equipped for epidemic control. It is worth noting that leaders must listen to PCPs’ concerns and encourage them to ask for help, instead of blaming or criticizing them. An array of feedback channels, such as listening sessions and email suggestion box, could be considered to make PCPs’ voice be part of the decision-making process.^33^

Second, the burden of unnecessary work could be reduced to maximize the capacity of PCPs during this turbulent time. PCPs wished to be freed from non-essential tasks and meetings to perform to their full potential and provide integrated care for residents in the community. It is advisable to consider innovative ways proposed by participants in our study to reduce workload and streamline procedures, such as rational workforce arrangement, effective internal coordination, and establishing an intelligent system of communication and surveillance.

Third, professional training could be provided to community workers to help facilitate their cooperation with PCPs. Current healthcare systems in many countries are under extreme pressure, and the use of community workers for the COVID­19 response would fill gaps in routine primary care.^34^ There is a potential to improve community workers’ capacity to deliver a wider range of care for the residents. For example, community workers receiving a basic training program might help PCPs manage older people in terms of drug delivery and collection of medical information, and this idea should be investigated in a future study.

Fourth, steps should be taken to build a more people-centered primary care system. Several long-standing limitations to China’s primary care system, particularly the shortage of professional human resources, substantially increased the difficulty of epidemic control in the community.^35^ PCPs in China are paid low wages and minimal benefits, receive inadequate training, and experience high rates of occupational burnout, which impede PCPs’ delivery of integrated and high-quality care.^36^ Therefore, the primary care system should ensure an adequate total income and strengthen the career development opportunities for PCPs.

## LIMITATIONS

There are several limitations in our study. First, the results may not be generalizable to other regions of China because we only interviewed PCPs in four provinces. Second, PCPs other than clinicians (e.g., nurse practitioners) were not included in our study because their scope of responsibilities was narrower than that for clinicians. Third, our study was not designed to compare the differences between urban and rural areas. Finally, we were unable to triangulate the results with those from other stakeholders, such as policymakers and community workers, but we will consider this in future studies.

## CONCLUSIONS

PCPs in China perceived a series of barriers in confronting the COVID-19 epidemic, which had positive and negative effects on their physical and mental health. Therefore, effective approaches are urgently needed to help PCPs overcome these barriers and work in an orderly and efficient manner. The current findings offer important lessons for policymakers and leaders for improving future control measures. In addition, they highlight the importance of developing the primary care system to strengthen preparedness and response to upcoming health challenges.

## Electronic supplementary material

ESM 1 (DOCX 23 kb)

## **Abbreviations**

PCPs: Primary care practitioners
PPE: Personal protective equipment
GPs: General practitioners
COVID-19: Coronavirus disease 2019

## References

[1] The Lancet (2020). Emerging understandings of 2019-nCoV. Lancet..

[2] Paules CI, Marston HD, Fauci AS (2020). Coronavirus Infections-More Than Just the Common Cold. JAMA..

[3] **World Health Organization.** Rolling updates on coronavirus disease (COVID-19). https://www.who.int/emergencies/diseases/novel-coronavirus-2019/events-as-they-happen. Published March 20, 2020. Accessed March 21, 2020.

[4] China Daily**.** Latest data on novel coronavirus. https://www.chinadaily.com.cn/china/special_coverage/2020latestdata. Published June 13, 2020. .

[5] **Bedford J, Enria D, Giesecke J, Heymann D, Ihekweazu C, Kobinger G, Lane HC, Memish Z, Oh M, Sall AA, Schuchat A, Ungchusak K, Wieler LH, WHO Strategic and Technical Advisory Group for Infectious Hazards.** COVID-19: towards controlling of a pandemic. Lancet. 395(10229):1015-1018. 10.1016/S0140-6736(20)30673-5. 10.1016/S0140-6736(20)30673-5 PMC7270596 32197103

[6] Gates B (2020). Responding to Covid-19—A Once-in-a-Century Pandemic?. N Engl J Med.

[7] **National Health Commission of the People’s Republic of China.** Nearly 4 million primary care practitioners are participating in prevention and control of the epidemic outbreak (in Chinese). https://www.sohu.com/a/373774455_374886. Published February 17, 2020. Accessed March 23, 2020.

[8] Li DKT, Zhu S (2020). Contributions and challenges of general practitioners in China fighting against the novel coronavirus crisis. Fam Med Com Health..

[9] Lim WH, Wong WM (2020). COVID-19: Notes from the Frontline, Singapore’s Primary Healthcare Perspective. Ann Fam Med..

[10] Kunin M, Engelhard D, Piterman L, Thomas S (2013). Response of general practitioners to infectious disease public health crises: an integrative systematic review of the literature. Disaster Med Public Health Prep..

[11] Kunin M, Engelhard D, Thomas S, Ashworth M, Piterman L (2015). Challenges of the pandemic response in primary care during pre-vaccination period: a qualitative study. Isr J Health Policy Res..

[12] Zhao Y, Zhang F, Ge X, Gong J, Li R, Wu D, Li X (2020). Survey on the Difficulties, Needs and Psychological Status of General Practitioners in Community-based Prevention and Control of the COVID-19 Epidemic. Clin Educ Gen Pract.

[13] Wong SY, Wong W, Jaakkimainen L, Bondy S, Tsang KK, Lee A (2005). Primary care physicians in Hong Kong and Canada—how did their practices differ during the SARS epidemic?. Fam Pract..

[14] Jaakkimainen RL, Bondy SJ, Parkovnick M, Barnsley J (2014). How infectious disease outbreaks affect community-based primary care physicians: comparing the SARS and H1N1 epidemics. Can Fam Physician..

[15] Verma S, Mythily S, Chan YH, Deslypere JP, Teo EK, Chong SA (2004). Post-SARS Psychological Morbidity and Stigma Among General Practitioners and Traditional Chinese Medicine Practitioners in Singapore. Ann Acad Med Singapore..

[16] DeJonckheere M, Vaughn LM (2019). Semistructured interviewing in primary care research: a balance of relationship and rigour. Fam Med Community Health..

[17] World Medical Association (2013). World Medical Association Declaration of Helsinki: ethical principles for medical research involving human subjects. JAMA..

[18] Creswell JW (2013). Research Design: Qualitative, Quantitative, and Mixed Methods Approaches.

[19] **Mertens DM.** Research and Evaluation in Education and Psychology: Integrating Diversity With Quantitative, Qualitative, and Mixed Methods. 3rd edition. Thousand Oaks, CA: Sage Publications; 2009.

[20] Bazeley P (2013). Qualitative data analysis: Practical strategies.

[21] Liu C, Wang H, Zhou L, Xie H, Yang H, Yu Y, Sha H, Yang Y, Zhang X (2019). Sources and symptoms of stress among nurses in the first Chinese anti-Ebola medical team during the Sierra Leone aid mission: A qualitative study. Int J Nurs Sci..

[22] Liu Y, Zhai Z, Hu D, Li M, Yang L, Xu F (2020). Qualitative study on psychological experience of front-line nurses combating new coronavirus pneumonia. Chin J Mod Nurs..

[23] Hsieh HF, Shannon SE (2005). Three approaches to qualitative content analysis. Qual Health Res..

[24] Uhl MC, Muth C, Gerlach FM, Schoch G, Müller BS (2018). Patient-perceived barriers and facilitators to the implementation of a medication review in primary care: a qualitative thematic analysis. BMC Fam Pract..

[25] Rimmer A (2020). Covid-19: GPs call for same personal protective equipment as hospital doctors. BMJ..

[26] Kamerow D (2020). Covid-19: Don’t forget the impact on US family physicians. BMJ..

[27] Adams JG, Walls RM (2020). Supporting the Health Care Workforce During the COVID-19 Global Epidemic. JAMA..

[28] Xiang YT, Yang Y, Li W, Zhang L, Zhang Q, Cheung T, Ng CH (2020). Timely mental health care for the 2019 novel coronavirus outbreak is urgently needed. Lancet Psychiatry..

[29] Lai J, Ma S, Wang Y, Cai Z, Hu J, Wei N, Wu J, Du H, Chen T, Li R, Tan H, Kang L, Yao L, Huang M, Wang H, Wang G, Liu Z, Hu S (2020). Factors Associated With Mental Health Outcomes Among Health Care Workers Exposed to Coronavirus Disease 2019. JAMA Netw Open..

[30] Kisely S, Warren N, McMahon L, Dalais C, Henry I, Siskind D (2020). Occurrence, prevention, and management of the psychological effects of emerging virus outbreaks on healthcare workers: rapid review and meta-analysis. BMJ..

[31] Liu S, Yang L, Zhang C, Xiang Y, Liu Z, Hu S, Zhang B (2020). Online mental health services in China during the COVID-19 outbreak. Lancet Psychiatry..

[32] Sibeoni J, Bellon-Champel L, Mousty A, Manolio E, Verneuil L, Revah-Levy A (2019). Physicians’ Perspectives About Burnout: a Systematic Review and Metasynthesis. J Gen Intern Med..

[33] Shanafelt T, Ripp J, Trockel M (2020). Understanding and addressing sources of anxiety among health care professionals during the COVID-19 pandemic. JAMA..

[34] Haines A, de Barros EF, Berlin A (2020). National UK programme of community health workers for COVID-19 response. Lancet..

[35] Yip WC, Hsiao WC, Chen W, Hu S, Ma J, Maynard A (2012). Early appraisal of China’s huge and complex health-care reforms. Lancet..

[36] Li X, Lu J, Hu S, Cheng KK, Maeseneer JD, Meng Q, Mossialos E, Xu DR, Yip W, Zhang H, Krumholz HM, Jiang L, Hu S (2017). The primary health-care system in China. Lancet..

