# Supplementary material for: Primary Care Practitioners’ Barriers to and Experience of COVID-19 Epidemic Control in China: a Qualitative Study
Source: J Gen Intern Med. 2020 Aug 31;35(11):3278–84. doi: 10.1007/s11606-020-06107-3 (PMC7458355; doi:10.1007/s11606-020-06107-3)
Supplement: Supplementary file 1 — (DOCX 23 kb) [file 11606_2020_6107_MOESM1_ESM.docx]

**Appendix 1.** Interview Guide

Thank you so much for agreeing to interview with me today. I express my love and care for you and your colleagues who are responding to the COVID-19 outbreak wholeheartedly. I appreciate hearing your story and perspectives about work in epidemic control. Please note the questions are not specific to you. There are no right or wrong answers, and you are not expected to answer in a certain way.

I hope that your experience and perspectives might inform policymakers and managers and improve countermeasures in managing major infectious diseases and professional training in the near future.

Now I begin recording and will write down some key words you mentioned.

**1 Could you please talk about your work responsibilities in epidemic control?**

- Which part of work do you think was well performed? why?

- Which part of work do you think was performed not very well? why?

- Which part of work do you think brought much pressure on you?

**2 What barriers and difficulties did you face in controlling the outbreak?**

- How did the barriers or difficulties affect your work?

- How did the barriers or difficulties affect your life?

- How do you evaluate your colleagues’ work?

- How do you evaluate other partners’ work? (e.g., managers, police officers, community workers)

- Have you tried to take any steps to overcome the difficulties you mentioned?

- Are things getting better now? What had been done for the barriers?

**3 What did you feel during the work of controlling the outbreak?**

- Have you experienced any positive or negative emotion? In which situations?

- Under what circumstances can your negative emotions or bad feelings be relieved?

- Have you tried to find external psychological support? Why or Why not?

**4 What do you think of the measures of epidemic control in primary care?**

- Have you thought about any measures to alleviate the barriers you encountered?

- What interventions can you think may improve epidemic control?

- What have you learned from participating in epidemic control?

| **ID** | **Sex** | **Age, y** | **Specialty** | **Practice setting** | **Area** | **Years in Practice** | **Administrative tasks** |
| --- | --- | --- | --- | --- | --- | --- | --- |
| P001 | M | 29 | Family physician | T | Shaanxi | 5 | No |
| P002 | M | 37 | Family physician | C | Guangdong | 15 | Yes |
| P003 | M | 34 | Family physician | C | Guangdong | 10 | Yes |
| P004 | F | 30 | Family physician | T | Shaanxi | 4 | No |
| P005 | F | 29 | Internist | T | Shaanxi | 3 | No |
| P006 | F | 29 | Family physician | C | Guangdong | 5 | No |
| P007 | M | 34 | Family physician | C | Zhejiang | 10 | No |
| P008 | F | 31 | Internist | C | Zhejiang | 6 | No |
| P009 | M | 37 | Family physician | T | Shaanxi | 10 | No |
| P010 | M | 38 | Family physician | C | Guangdong | 17 | Yes |
| P011 | F | 37 | Family physician | C | Zhejiang | 15 | No |
| P012 | M | 43 | Internist | C | Guangdong | 23 | Yes |
| P013 | F | 46 | Family physician | C | Zhejiang | 25 | No |
| P014 | F | 37 | Pediatrician | C | Guangdong | 18 | No |
| P015 | F | 37 | Family physician | C | Guangdong | 14 | No |
| P016 | M | 42 | Surgeon | C | Zhejiang | 20 | Yes |
| P017 | M | 34 | Family physician | C | Guangdong | 11 | No |
| P018 | F | 35 | Family physician | C | Guangdong | 11 | No |
| P019 | F | 36 | Internist | C | Zhejiang | 10 | No |
| P020 | M | 38 | Surgeon | C | Zhejiang | 13 | No |
| P021 | M | 38 | Family physician | T | Hunan | 12 | No |
| F = female; M = male; T=Township health center; C=Community health center | | | | | | | |

**Appendix 2.** Participant characteristics

**Appendix 3 COREQ Checklist**

| **Topic** | **Item No.** | **Reported information** | **Reported on page No.** |
| --- | --- | --- | --- |
| **Domain 1: Research team**  **and reﬂexivity** | | | |
| *Personal characteristics* | | | |
| Interviewer/facilitator | 1 | Zhijie Xu; Yuanqu Ye | 8 |
| Credentials | 2 | M.B.B.S. (Z.X.); M.D. (Y.Y.) | 1 |
| Occupation | 3 | Both are general practitioners | 9 |
| Gender | 4 | Both are male | 9 |
| Experience and training | 5 | Both received training of qualitative study | 9 |
| *Relationship with participants* | | | |
| Relationship established | 6 | No participant had worked together with the investigators | 8 |
| Participant knowledge of the interviewer | 7 | Participants knew the interviewers prior to the interview | 8 |
| Interviewer characteristics | 8 | Not reported | N/A |
| **Domain 2: Study design** | | | |
| *Theoretical framework* | | | |
| Methodological orientation and Theory | 9 | Thematic content analysis. | 9 |
| Participant selection | | | |
| Sampling | 10 | Purposive sample in four provinces | 8 |
| Method of approach | 11 | WeChat | 7 |
| Sample size | 12 | 21 | 9 |
| Non-participation | 13 | three family physicians refused the participation because they were not responsible for tasks in epidemic control. | 8 |
| *Setting* |  |  |  |
| Setting of data collection | 14 | Phone call | 7 |
| Presence of non-participants | 15 | No | N/A |
| Description of sample | 16 | 14 family practitioners, 4 internists, 2 surgeons and 1 pediatricians; 10 were women and 5 participants undertook administrative tasks in their medical practice. The mean age of participants was 36 years (range: 29-46 years), and the mean duration of practice was 12 years (range: 3-25 years) | 9 |
| *Data collection* | | | |
| Interview guide | 17 | It was adapted from relevant qualitative studies and was refined through pilot interviews with 3 PCPs | 8 |
| Repeat interviews | 18 | No | 8 |
| Audio/visual recording | 19 | Audio recording was used | 9 |
| Field notes | 20 | Both interviewers made field notes during interview when necessary | 9 |
| Duration | 21 | a mean of 34 minutes (range, 30-45 minutes) | 7 |
| Data saturation | 22 | Yes | 8 |
| Transcripts returned | 23 | No | 9 |
| **Domain 3: analysis and**  **ﬁndings** | | | |
| *Data analysis* | | | |
| Number of data coders | 24 | two investigators identified major themes and subthemes via thematic content analysis | 9 |
| Description of the coding tree | 25 | Not reported | N/A |
| Derivation of themes | 26 | Themes were derived from data collected | 9 |
| Software | 27 | MAXQDA (version 2018.1.1) | 9 |
| Participant checking | 28 | 3 randomly selected participants agreed with the themes without modification. | 9 |
| *Reporting* | | | |
| Quotations presented | 29 | Yes | Results |
| Data and ﬁndings consistent | 30 | Yes | Results |
| Clarity of major themes | 31 | Major themes resulting from the interviews were outlined in Results. | Results |
| Clarity of minor themes | 32 | Minor themes resulting from the interviews were outlined in Results. | Results |
